# Management of Medical Emergencies on Psychiatry Inpatient Floors: A Novel Simulation-Based Curriculum for Psychiatry Residents

**DOI:** 10.7759/cureus.92872

**Published:** 2025-09-21

**Authors:** Jason G Emsley, Cindy Shearer, Donna Warren, John Ross

**Affiliations:** 1 Emergency Medicine, Dalhousie University, Halifax, CAN; 2 Medical Education, Dalhousie University, Halifax, CAN

**Keywords:** curriculum development and evaluation, interprofessional education and collaboration, psychiatry training programs, resuscitation, simulation in medical education, teaching in emergency medicine

## Abstract

Introduction

For most psychiatry residents, training in managing the early phases of acute medical emergencies is limited to exposure during medical school and junior off-service rotations. Patients with psychiatric illnesses, however, often have higher rates of medical comorbidities than their age-matched controls. We developed a half-day medical refresher and simulation curriculum to improve the confidence and efficacy with which psychiatry residents can manage medical emergencies on inpatient floors.

Methods

Based upon a detailed needs assessment conducted with psychiatry residents at various stages of their training, we developed nine clinical scenarios that could be encountered by psychiatry residents managing inpatient units. These included: shortness of breath; sepsis, acute coronary syndrome; cardiac arrest; seizure; overdose; laceration; asphyxiation from hanging; and smoke inhalation. Training sessions included basic skills stations (such as how to obtain vitals or perform bag mask ventilation). Participants completed anonymous surveys prior to the sessions to provide information on their location and level of residency training and their experience with managing medical emergencies. Pre- and post-training surveys assessed participants’ confidence levels with various basic procedural skills and general emergency management. Focus groups were performed to obtain qualitative data about participants’ experiences and opinions about the simulation scenarios and training session.

Results

Level of experience varied among participants with respect to exposure to medical emergencies. Most respondents reported a strong desire to have training sessions on these topics. Reported confidence levels increased across multiple domains. Further, residents in general expressed high satisfaction with the scenarios and skills sessions. Residents also identified topics for further medical simulations.

Discussion

We developed a half-day simulation curriculum focused on essential emergency skills and initial management of a variety of plausible scenarios that could be encountered on a psychiatry inpatient unit. This curriculum could be easily integrated and regularly run as part of any psychiatry residency program. Most importantly, this program may greatly enhance the confidence and efficacy of psychiatry residents faced with managing the initial phase of medical emergencies.

## Introduction

Psychiatry residents in training programs across North America gain exposure to basic medical management of sick patients during medical school (through clinical rotations and courses such as Basic Life Support (BLS) and Advanced Cardiovascular Life Support (ACLS), as well as during their early “off-service” portions of their training. In subsequent years of training, however, their focus by necessity centres around psychiatric assessment, management, and scholarship. This focus away from basic "medical" management may leave many residents feeling that their ability to handle emergency medical situations may significantly decline. These changes are not because of a lack of interest. Indeed, it was the expressed desire among many psychiatry residents in our institution that inspired the development of the simulation curriculum we will describe below. 

There are few studies that illustrate the use of simulation for psychiatry-related emergencies. Some of these are aimed toward undergraduate medical education, and the majority of studies only address presentations such as lithium toxicity, overdoses, altered mental state, or delirium tremens, rather than acute “non-psychiatric” complaints such as chest pain, shortness of breath, or trauma [1-4]. One well-executed study did look at several medical emergencies such as respiratory arrest, hypoglycemia, choking, and hanging, with a significant majority of the participants being nurses or healthcare assistants [5]. Another looked at training using an Essential Life Support (ELS) course and found subsequent improvements in self-reported confidence, knowledge, and attitudes in mental health providers [6].

Many patients with mental illness are often concurrently dealing with other physical diseases. Indeed, the burden of physical illnesses on patients with psychiatric diagnoses is greater than that in patients without psychiatric illness [7-10]. Patients on psychiatric inpatient units, just like those on more traditional “medical” floors, may develop significant presentations such as acute coronary syndromes, exacerbations of chronic obstructive pulmonary disease (COPD), and life-threatening sepsis. In addition, psychiatric inpatients may endure self-harm due to overdoses and trauma, necessitating immediate recognition and management by their treating team.

With the overarching goal of improving management of medical emergencies for psychiatric inpatients, our objective here was to improve the confidence with which psychiatry residents manage the initial phases of a medical emergency. To do so, we first performed a needs assessment of psychiatry residents at various stages of their training. Based upon this analysis, we then developed a novel and easily implemented half-day simulation curriculum that used nine scenarios that simulated a variety of medical emergencies that residents could encounter while managing inpatient psychiatric units. Using Kirkpatrick’s model of evaluation, especially focusing on the initial stages of reaction, learning, and behavior, we assessed the efficacy of the training and simulation program [11] using both quantitative and qualitative measures. Specifically, we performed pre- and post-simulation comparisons to assess changes in self-rated confidence in management of medical emergencies, as well as conducted in-depth focus groups to explore the perceived efficacy of the simulations to improve these critical skills in psychiatry residents.

## Materials and methods

Goals

The training session and scenarios we developed were specifically designed to 1) review the medical burden of disease on psychiatric patients; 2) identify “sick” versus “not sick” patients (a foundational principle in Emergency Medicine); 3) perform initial steps in stabilizing and managing a variety of emergent presentations (the “ABCs”); 4) manage team dynamics during an acute medical event; and 5) have a safe approach to bringing in additional resources (first responders, code teams, and consultants).

Development

Based on these goals, we developed the simulation curriculum by first implementing a needs assessment for psychiatry residents at our institution. The assessment was circulated in the form of an anonymous online survey, distributed to residents in all five years of Dalhousie University’s FRCPC program in Psychiatry. Basic demographic data included year of residency and whether they had prior experience in managing medical emergencies during any of their psychiatry rotations. Respondents were asked about their level of interest in a half-day curriculum, and if they had suggestions for specific medical cases they would want to see covered. We took all of the needs assessment feedback into account as we developed cases most germane to the past and possible future clinical experiences of psychiatry residents.

All participants were expected to have at least some training in initial management of medical emergencies (even if this was entirely within their medical school training). We did not make it a requirement that residents had completed BLS, ACLS, Pediatric Advanced Life Support (PALS), or other courses within a set period of time. However, completion of BLS and ACLS courses are required at our institution prior to medical school graduation, and having these or similar certifications is common in many medical schools in Canada and the USA. Facilitators were expected to have experience in emergency medicine and to be familiar with simulation-based learning and supportive debriefing.

Prior to implementing the simulation scenarios (described below), we performed “test runs” of all the simulations with a volunteer group of emergency medicine residents at various stages of training. We used this group as they are highly familiar with simulation as part of their training and were able to provide extremely useful feedback on the feasibility and level of complexity of the cases.

Equipment

We purposely used equipment that would be identical to or as similar as possible to that which would be found in the emergency kits/“crash carts” found in our institution’s main psychiatric inpatient facilities. The list of equipment is outlined in Table 1.

**Table 1:** Equipment List AED: automated external defibrillator, ASA: acetylsalicylic acid, CPR: cardiopulmonary resuscitation, PEEP: positive end-expiratory pressure

Mannikin, examination, and monitoring equipment
low or high fidelity mannikin (we used a Laerdal SimMan Essential system)
stretcher
johnny shirt, sheets, blanket, pillow
street clothing for mannikin, as appropriate for each simulated scenario
stethoscope
Sim defibrillator/AED with defibrillation pads
portable O2 sat monitor
blood pressure cuff
glucometer
thermometer
penlight
Airway-related equipment
nasal prongs
non-rebreather mask
bag mask ventilator (BMV) with masks
PEEP valve
Oxygen tubing
O2 tank
nasopharyngeal airways (NPAs) (several sizes)
oropharyngeal airways (OPAs) (several sizes
Simulated medications
ASA
naloxone (Narcan) for intramuscular (IM) administration
benzodiazepines for IM administration (e.g. lorazepam, midazolam, valium)
anti-psychotics for IM administration (e.g. olanzapine, haloperidol)
nitroglycerin spray (sublingual)
glucagon (oral)
10 cc syringes
blunt fill needles
needles for simulating IM injection
Miscellaneous
gauze
cling
abdominal “trauma” pads
medical tape
scissors
cervical collar
CPR backboard
cutting device for belts, ropes etc.
disposable gloves (various sizes)
disposable gowns
masks
eye protection

For the majority of the simulation scenarios, we used a high-fidelity mannikin (one with which we normally do high-level resuscitation scenarios for emergency medicine residents, staff, nurses, and paramedics). While this mannikin has the ability to produce realistic sounds (such as coughing, wheezing, vomiting etc.), and has realistic and adjustable lung sounds, heart rate, pupil size, and airway features, we must emphasize that a high-fidelity mannikin is not necessary for the successful implementation of these scenarios. Each scenario has been written for use with any “basic”, low-fidelity mannikin.

Personnel

The course was facilitated by two of the authors (JE and DW): at the time of course implementation, JE was a senior resident in the Canadian five-year FRCPC Emergency Medicine program, with training in simulation for medical students and residents, and DW was a Critical Care Paramedic (CCP) who oversaw the emergency medicine simulation program at our institution. We recommend that with a learning group size of five to ten learners, the course could be run by two individuals with experience in emergency medicine and running medical simulations and debriefing. Some of the simulation cases, as described below, benefit by employing at least one or two of the participants to act as embedded participants during the case.

Implementation

As described above, this was a focused simulation curriculum directed toward improving the skills and confidence of Psychiatry residents in managing medical emergencies on psychiatric inpatient units. While residents were our primary users of the curriculum, we also believe that these situations could be easily adapted for use with medical students, hospitalists, psychiatry staff, and nurses. Indeed, these scenarios would likely be beneficial for future interdisciplinary simulation training sessions.

The training sessions we ran took approximately three hours. Each session included the following six components.

First, we gave introductory remarks and the rationale for having the sim sessions.

Second, we had an orientation to simulation in general. We specifically stressed to participants that the simulations were a chance to practice managing medical emergencies, and that these were learning, rather than testing, opportunities. As residents were not particularly used to this form of simulation in their training, we made it very clear that this was a safe learning environment, free from judgment, and that their performance during the cases was not part of any evaluative process. We felt that these introductory discussions helped to set the tone for a lower-stress, productive learning environment for the participants. We also briefly explained how the mannikin worked, where the equipment could be found, and reviewed what monitoring equipment was available.

Third, we gave a brief refresher on use of equipment. We did a short (~10 to 15 minute) refresher on placement of an oxygen saturation probe, obtaining a manual blood pressure reading, placing nasal prongs and non-rebreather masks, and performing effective bag mask ventilation. We reviewed effective cardiopulmonary resuscitation (CPR) techniques, as well as the importance of team safety and the use of personal protective equipment such as gloves, gowns, and masks. Finally, we reviewed the contents and organization of crash carts that were available for use at our institution’s psychiatry inpatient units.

Fourth, we ran a total of nine simulations, each lasting approximately 10 to 15 minutes, plus five to 10 minutes to debrief each case after it happened. The case types are listed below, in the order in which they ran. A brief description of each case is provided in Table 2.

**Table 2:** List of Simulation Scenarios COPD: chronic obstructive pulmonary disease, NYD: not yet determined

Scenario	Description
Shortness of breath	A 74-year-old admitted with schizoaffective disorder develops an acute exacerbation of her COPD
Sepsis	A 52-year-old admitted with a major depressive episode, with worsening cellulitis
Acute coronary syndrome	A 59-year-old admitted with worsening obsessive-compulsive disorder and generalized anxiety disorder, who has developed an acute coronary syndrome
Cardiac arrest	A 48-year-old admitted with chronic schizophrenia, who has a cardiac arrest
Seizure	A 27-year-old admitted with psychosis NYD develops a generalized tonic-clonic seizure
Opioid overdose	A 24-year-old with a history of substance abuse returns from an unescorted pass and overdoses on opioids
Laceration	A 19-year-old admitted following a recent suicide attempt cuts her wrist, resulting in significant arterial bleeding
Asphyxiation	A 29-year-old admitted with a major depressive disorder is found hanging by a belt
Mattress fire	A 71-year-old admitted with an exacerbation of his chronic schizophrenia falls asleep while smoking and inadvertently sets fire to his mattress

We began with the fairly simple case of shortness of breath, as this helped to reinforce basic assessment and fundamental airway and breathing management skills. Many of the subsequent simulations built upon these general airway and breathing management skills. All of the simulated cases used in this curriculum are found in Appendix 1.

Fifth, we debriefed each case immediately after it occurred. Typically, one member of the group would give a very brief summary of the case, followed by inviting the team leader to share how they felt the case went. Everyone was encouraged to provide constructive input. We would review separately, with each case, specific technical points including medical management, procedures (e.g., bag mask ventilation), and what the definitive steps would be for that patient’s care. We would then, as a group, with guided facilitation, review and highlight any key communication, team dynamic, and process issues. In all cases, we would specifically highlight team communication in the context of the type of environments in which residents might in the future manage these or similar cases. Finally, each session concluded with an open-ended debrief period, where participants could discuss any aspect of specific cases and their overall experiences. We also used this time for participants to discuss team dynamics and communication, and to consider what might be the barriers to providing effective care in psychiatric inpatient units. General, informal feedback about the cases and the session was also sought as part of the debrief. Note that the case-specific debrief points are included at the end of each case, as are the guiding question for the general (post-session) debrief.

Sixth, building upon the sessions outlined below, we have gone on to run further training sessions for additional groups of residents, psychiatry floor inpatient hospitalists, along with interdisciplinary sessions including hospitalists, residents, and nurses.

Evaluation

Using Kirkpatrick's model, we evaluated the efficacy of this half-day simulation curriculum using a variety of quantitative and qualitative measures to assess residents' initial reaction, learning, and behavior. Prior to the course, respondents either filled out a paper or an online survey about their level of residency training, as well as additional demographic data related to location of medical school training (e.g., Canada or US). Residents were also polled as to whether they had completed a rotation in Emergency Medicine, and whether they had taken specific courses in medical emergency management such as BLS, ACLS, or PALS. We also inquired as to whether residents had encountered medical emergencies in their psychiatry training to date, and their level of comfort dealing with those cases. Further, we asked whether respondents were familiar with the location of crash carts and airway kits in the locations where they had prior psychiatry clinical rotations or on-call experiences.

Respondents also completed a pre-session survey of medical preparedness using a modified Likert scale [12], wherein they rated their level of confidence in, for example, obtaining a set of vitals, applying nasal prongs, performing effective chest compressions, and directing team members during a medical crisis. A select group of residents also responded to a post-session survey, which specifically measured their confidence levels in performing these tasks after having been part of the simulation curriculum. We examined for differences in confidence scores for each of the skills domains, and also (despite the low sample sizes of respondents (N=7 in pre-test and N = 2 in post-test) calculated for possible statistical significance. For this we employed Welch’s t-test for independent samples, with significance set at p < 0.5.

For qualitative analysis, our needs assessment first asked open-ended questions of respondents as to how useful they felt a dedicated simulation curriculum would be. After running the sessions, one of us (CS) performed focus group sessions with residents. From these approximately one-hour sessions, we identified common themes related to how psychiatry residents feel about managing medical emergencies, their sense of confidence in these types of cases, and how they felt the curriculum would increase their skills and confidence in managing these and similar cases in future clinical encounters.

## Results

Needs assessment

To guide the development, testing, implementation, and assessment of the simulation curriculum, we performed an anonymous, online, survey-based needs assessment. A total of 27 residents from the FRCPC Program in Psychiatry responded to the survey, and there was an essentially even distribution across all five years of the program. Nineteen of the respondents attended a Canadian medical school, six attended medical school outside of Canada or the US, and two preferred not to answer. The majority (> 95%) of respondents had completed residency rotations in psychiatric acute care prior to completing the needs assessment. Approximately 18% had completed rotations in a forensic psychiatry setting, and slightly over 50% of the respondents had completed a rotation in pediatric psychiatry. A total of 25 of the 27 respondents had completed a rotation in emergency medicine. All of the respondents had taken BLS training, with approximately 40% of them completing this training within two years of the needs assessment. Similarly, all of the respondents had taken ACLS, with approximately 45% of them completing this training within two years of the needs assessment. Three of the respondents had completed a PALS course.

Approximately 65% of respondents had participated in the management of a medical emergency on a psychiatric unit. Approximately 65% of those respondents had participated in more than one emergent incident. A total of 88% of those respondents stated that their experiences with medical emergencies on psychiatric units occurred while they were on call. Only one resident felt comfortable managing the medical emergency prior to the arrival of additional resources arrived (such as a senior colleague, hospitalist, paramedics, ICU, or a code team), while four stated they felt “somewhat comfortable.” The remainder of the respondents reported that they felt either “somewhat uncomfortable” (six respondents) or “uncomfortable” (seven respondents).

We asked about residents’ familiarity with the location of critical resuscitation equipment on the floors in which they were performing inpatient acute psychiatric care rotations. Six of the 27 respondents (22%) were able to identify the location of the crash carts on the acute care floors and only two of the 27 respondents (7%) reported that they knew the location of airway equipment.

Finally, we asked respondents if they would be interested in attending an informal, interactive session reviewing basic medical management skills and participating in simulations focused on emergencies that may occur on a psychiatric inpatient unit. The vast majority (96%) of respondents indicated a strong willingness to attend.

Quantitative results: pre- and post-training assessments (confidence scales)

We employed a mixture of quantitative and qualitative measures to assess the efficacy of the sessions. Prior to one of the training sessions, we asked residents to rate their level of confidence in various domains of assessment and management of acutely ill patients. Questions included, for example, their initial (pre-simulation session) level of confidence in the following domains: recognizing that a patient may need more definitive care; obtaining a set of vitals; locating emergency equipment; assisting a patient with respiratory compromise; initially managing a major laceration; performing effective CPR; coordinating a resuscitation, and communicating with responding services. A group of residents received these pre- and post-session surveys, but unfortunately the sample sizes were too low for us to confidently determine statistical significance (an n of 8 for the pre-session questionnaire and an n of 2 for the post-session questionnaire). Respondents were asked to rate their level of confidence within each domain, with a score of zero indicating not confident at all to 10 indicating completely confident. The mean and standard deviations for those scores are summarized in Table 3.

**Table 3:** Pre- and Post-test Confidence Scores Welch's t-test for independent samples (two-tailed); Significance set at p< 0.05.

	Pre-test		Post-test			
	Mean	SD	Mean	SD	t-statistic	p-value
Recognizing that a patient will need medical care beyond that available within the psychiatric inpatient unit (e.g., transfer to ED or to Internal Medicine floor)	6.86	1.57	9.50	0.71	-3.53	0.023
Obtaining a set of vitals (including glucose)	7.14	2.19	9.50	0.71	-2.31	0.082
Locating equipment necessary for airway management	4.00	2.38	8.50	0.71	-3.36	0.077
Applying nasal prongs and oxygen	6.71	2.75	10.00	0.00	-2.32	0.053
Applying face mask and oxygen	6.43	2.70	10.00	0.00	-2.36	0.05
Positioning a patient to optimize oxygenation and ventilation	6.00	2.82	9.50	0.71	-2.5	0.087
Effectively using a bag mask for ventilation (aka "bagging")	6.14	2.12	9.00	0.71	-2.71	0.07
Insertion of airway devices such as an oropharyngeal airway (OPA) to help open up the airway for better bag mask valve oxygenation	4.29	2.87	9.00	0.71	-2.57	0.123
Initial treatment of a major laceration (e.g., application of appropriate pressure dressings)	5.00	2.58	6.50	0.71	-0.97	0.414
Performance of effective chest compressions	6.00	3.06	9.00	0.00	-2.42	0.074
Running a code for a cardiac arrest before a code team arrives	2.43	1.90	8.50	0.71	-5.66	0.059
Directing team members (nurses, junior house staff, ward aides, security) during a medical crisis)	3.71	1.70	8.50	0.71	-4.75	0.081
Communicating and liaising with EMS dispatch about a patient requiring transport	5.14	1.77	9.50	0.71	-4.17	0.097
Communicating with consultants (e.g., emergency physician, internal medicine physician) about the medical emergency	6.43	1.13	9.50	0.71	-4.34	0.096

Given the lower sample sizes, we cannot draw definitive conclusions from the pre- and post-session confidence scores, yet the preliminary trends suggest potential areas of improvement. Reported confidence levels appeared to increase in several key domains, particularly with respect to recognizing that a patient was unwell and likely needed more urgent and definitive medical care as well as in identifying where one could find critically needed equipment. Further, increases were also observed in domains related to the management of respiratory compromise (applying nasal prongs or face masks, positioning a patient’s airway, or providing effective bag mask ventilation). In addition, respondents reported improved confidence in performing effective CPR and communication skills.

Qualitative results: post-training assessments (focus groups)

We conducted two post-session focus groups, each lasting about 60 to 90 minutes. Attendees were all Psychiatry residents at various levels of their residency training. There was a total of seven participants, with three in one of the sessions and four in the other. The sessions were led by CS, who was not directly involved in either organizing or running the simulation scenarios. Questions were aimed at understanding participants’ prior experiences with medical emergencies on psychiatric inpatient floors; their level of experience with medical simulations; the quality of the simulation scenarios; the overall quality of the educational environment; any other suggestions and general comments for future scenarios. Each of these themes, along with samples of representative comments from participants, is summarized in Table 4.

**Table 4:** Focus Group Themes and Representative Comments

Prior experiences and challenges
“I begged the anesthesia resident to help” with a patient with severe pneumonia
“These situations often arise when there is no one else around…”
The nurses “are not used to dealing with these situations either.”
“During the day you are more likely to get help quickly and easily.”
“We have not been doing this for probably a couple of years but we have done this. These simple things we can manage.”
“I think working people up when they’re not really that sick has been okay but it’s when you really get to the crunch and you need some support.”
“We need to improve our confidence so we can actually know to ask that we do need their (consultant’s) help…”
“And it’s hard to be comfortable because we’re in Psychiatry and we don’t do a lot of hands-on medicine. It’s been a couple of years out since we had to do any of that… We’ve been in situations in the emergency department where you’re surrounded by people who are confident and comfortable. So here you are now in the middle of the night by yourself. You know, no one is confident and comfortable, least of all you in your own skills.”
“…if someone crashes on one of our floors someone has to have the presence of mind to grab the elevator, ride the elevator up, negotiate with one of the nurses to have their crash cart, take the crash cart, take it down to one of us who is going to be bewildered…”
“We can’t change the system but we can change ourselves.”
Prior simulation experience
“People who get sim are in medicine (or in) emergency or ICU. There’s nothing like that in Psychiatry. So you might get it if you’re in one of those rotations in your first or second year but otherwise we’re not getting it.”
(Residents mainly get) “(s)imulated patients yes but not medical issues.”
Residents are often taught “Communication skills and things relevant to Psychiatry.”
Quality of the simulated scenarios
The scenarios are “(g)reat. They are the most common medical emergencies. A couple of them are very psychiatry-oriented: suicide, cutting, and the like. These are the things we can see.”
“Very realistic scenarios that we may come across.”
“The cases were all pretty pertinent. Things we probably see on the unit. The kind of comorbidities we see with our patients.”
“To a certain extent, making the diagnosis less clear would have some value.”
“I thought they were well balanced. They were acute. That’s good because I think when we have something less acute we’re better able to handle it… because you have more time you can think it through, you can look it up. So, since we only had a certain amount of hours (for the simulation session) to hit the highpoints where things can go bad and go bad quickly – I think that was a better use of time.”
“I think it’s applicable to Psychiatry and what we do. In medical school you get a lot of book knowledge and you know how to manage your cases overall but in these scenarios, (they teach) this is where you stop and this is where you hand over the patient for more further management.”
“And life skills too. Sometimes you’re on a plane or something and someone says… ‘is there a doctor on the plane?’ and… we’re doctors but I’m a doctor that hasn’t touched somebody in a really long time. As far as managing or doing CPR or any of that kind of stuff… this is really great to refresh that too.”
The educational environment
“(The instructors didn’t) go down ACLS or any of that which is great because we aren’t actually going to go down those algorithms. It’s just never going to happen. We don’t have the stuff. We can’t do it so why learn it? Why spend time learning that? We should focus on the things that we’re actually going to do which I felt like (the instructors) did today.”
“I feel like (the instructors didn’t) mock us at all if you didn’t know how to put on something or do something…”
“I don’t think (the instructors) assumed that because we are doctors we should know certain things. I feel like (the instructors) appropriately determined what our knowledge or what our experience has been and adapted (the sessions) perfectly.”
Changes in confidence level
“I feel like I’m ready.”
“…this kind of session really boosts the confidence that we have things that we can do and we should do…”
“(I)t’s really excellent that they’re sharpening our skills but it’s not just our skills that need to get sharpened.”
“… we need help and if (we) can feel comfortable providing help that is important.”
Suggestions for future sessions
(Having sessions that are) “interdisciplinary might be really good.”
“Sessions like this one are good but it should be at least once a year in order to maintain skills.
(Additional scenarios could include “different toxidrome(s), as well as withdrawals (and) falls” (as well as) “serotonin syndrome or NMS (neuroleptic malignant syndrome)”

With respect to prior experiences with medical emergencies on psychiatric inpatient floors, residents were asked to discuss any challenges they faced. How supported did they feel? What were the main barriers to providing care for the unwell or critically ill patient? Residents described situations where equipment, such as a Code Cart, could not be found, as well as situations where they had to “beg” consultants to provide assistance. In other cases, they felt well supported by consulting services. Participants also described how the time of day and the prior experience/skill sets of the nurses influenced how well they felt a case could be managed. Participants identified general “systemic” or flow issues, such as the lack of clearly accepted algorithms for transfer or escalation of care. Several residents also pointed out that, even though there are barriers, the sessions provided may substantially improve their confidence in effectively advocating for unwell or critically ill patients.

Participants were asked to reflect on the overall quality of the simulations, including how realistic and germane they felt these scenarios were. Participants described the scenarios as being very applicable to their practice, even if many of the cases would not occur frequently. Several respondents also added that having a “psychiatry spin” to the cases was helpful. Some comments identified that, while these scenarios are useful, they might also benefit from more vague presentations, such as headache or non-specific abdominal pain. Participants also felt that future sessions should incorporate multi-disciplinary teams of residents, nurses, and hospitalists. Further, a few participants suggested that future sessions could be held on one of the wards, or at least in a venue that recreated the space of a typical psychiatric inpatient room.

We asked participants to reflect on the overall quality of the educational environment. Participants described the learning environment as supportive and constructive. Several felt that the pragmatic approach, focusing on what would be germane to their situation and skill level, was ideal, and they appreciated that the scenarios did not go deep into the pathophysiology of any specific case. Participants also noted that they very much appreciated receiving “take home” messages about specific cases or skills. Finally, participants felt that keeping the groups to a total of no more than four to five learners was ideal, and gave each of them the chance to practice and work on their various skills.

Participants were then asked how much they felt these simulation sessions would improve their confidence in managing medical emergencies in the future. How did their overall confidence improve? What specific technical skills do they now feel were refreshed or enhanced by the sessions? Participants pointed out that they felt their skill levels and confidence in those skills were improved by attending the sessions. Several stated that their confidence in the overall management of a medical emergency had been improved, along with their confidence with a variety of hands-on skills, such as managing airway and breathing issues, or performing effective CPR. Further, participants commented that the sessions enhanced their confidence in being able to manage the team performing initial assessment and management of a sick patient.

Finally, residents were asked to share general suggestions about how we could improve future sessions. A few suggested that it would be helpful to have sessions to improve the confidence of the rest of the treatment team, especially nursing staff. One participant suggested that we could have some scenarios in which the presentations are less obvious. Further, a few participants suggested that, in addition to the opioid overdose scenario from these sessions, we add other toxidromes such as serotonin syndrome or neuroleptic malignant syndrome. Some participants also asked if there could be refresher sessions on suturing, as well as performing a focused physical examination. Finally, participants felt it would be helpful to integrate this type of simulation scenario into the annual teaching schedule so that skills could be regularly assessed and refreshed.

## Discussion

Limitations

While this curriculum appears to be a useful addition to psychiatry residency training, there are some limitations with how we were able to assess this intervention's efficacy. This was a single-centre study, and only involved residents from a single cohort. Further, our study focused mainly on resident physicians, and while we did go on to deliver interdisciplinary sessions and a session for hospitalists, the feedback we present here comes essentially from residents. Further, there is likely considerable heterogeneity among any multi-year resident cohort with respect to their level of experience in dealing with both simulated and real medical emergencies. With respect to our quantitative and qualitative analyses, our sample sizes (particularly for the focus groups) were small. Further, the response rates in some cases were low. There is therefore obviously a chance that the views expressed in our relatively small focus groups do not fully characterize all the possible opinions about the effectiveness and structure of the curriculum in general or the individual simulations in particular.

Another limitation of our study is that it does not consider how well these skills, or the level of confidence engendered by sim practice, are retained over significantly longer periods of time. Many participants identified that they would like to have this simulation curriculum delivered on a regular basis, and if this becomes possible, then further studies could be extended to focus on the fourth phase of the Kirkpatrick model, and examine retention of confidence and skill levels [11]. Further, we used self-reports of efficacy, and there is a possibility that there may be discrepancies between self-reported and independently assessed measures of efficacy [13]. Finally, as with many other educational initiatives, the ensuing COVID-19 pandemic made it very difficult to work at making this type of session a regular event. However, as pandemic-related restrictions on the running of education sessions, including simulation, have abated, there remains an opportunity to deliver this curriculum to a new cohort of psychiatry residents.

Strengths and future directions

We developed a novel simulation curriculum that can easily be scaled for psychiatry residency programs of various sizes, keeping in mind our suggested instructor-to-participant ratios. In developing and implementing this focused simulation curriculum. We identified several key principles to enhance its delivery. For example, when delivering these sessions to multiple groups of residents (along with various interdisciplinary groups and hospitalists), we found that the simulation scenarios run most effectively and efficiently if we begin with a brief overview of essential skills. This included how to quickly assess the sick versus not sick patient, obtaining vital signs, discussing a basic approach to providing respiratory support, and various procedural skills, particularly how to apply nasal prongs or face masks, performing bag-mask ventilation, and how to do effective CPR.

For all of these sessions we stress the importance of setting a non-judgmental tone, being mindful that many of the participants may have had very little recent simulation experience with acute medical cases. We aim to make the cases as interactive as possible, and found that residents embrace the opportunity to play different “roles” within the scenarios. We also discuss the importance of seeking help early, and discuss whom to call, depending on the institutional protocols. Giving the participants a chance to practice talking to a consultant or EMS provider is also highly important, and something many of them found particularly helpful. We also found that, in future sessions, we could add more toxicology cases, such as those focusing on serotonin syndrome and neuroleptic malignant syndrome. Finally, we also found it extremely helpful to provide an opportunity at the end of the session for participants to have, if they wish, further practice with the skills used throughout the sessions, as well as to have a brief discussion about how they can take these skills going forward to their next call shifts. Most importantly, we deliver all of these aspects of the short curriculum through the lens of improving confidence for residents in being able to provide optimal care for the population they serve.

## Conclusions

While it has been shown that mental health delivery simulations are highly effective for psychiatry residents, there are relatively few studies focusing on simulating medical emergencies in psychiatry patients. This novel, half-day curriculum provides a basic refresher in general clinical skills for psychiatry residents and other team members, aimed at improving the confidence and skills with which psychiatry residents can respond to and manage the initial phases of acute medical emergencies on psychiatric inpatient units. To our knowledge, no such standalone, sim-based curriculum exists. Based upon both quantitative and qualitative feedback, this short program may be of benefit to current and future psychiatry residents, hospitalists, nurses, and other allied health professionals.

## Appendices

Jason G. Emsley Appendix- Management of medical emergencies on psychiatry inpatient floors: a novel simulation-based curriculum for Psychiatry Residents-Psychiatry Simulation Scenarios.pdf

drive.google.com/file/d/1H4NYcKw4mQKSeTuIYs1Z8sTkfToL-8Bm/view?usp=sharing
